# Supplementary material for: Survival and Prognostic Factors of Ultra-Central Tumors Treated with Stereotactic Body Radiotherapy
Source: Cancers (Basel). 2022 Nov 29;14(23):5908. doi: 10.3390/cancers14235908 (PMC9737655; doi:10.3390/cancers14235908)

Supplementary Materials Table S1. Prognostic factor at multivariate analysis.

Covariate	HR (95% CI)	p value
Gender	0.6 (0.4-1.0)	0.054
Male (71)		
Female (51)		
Age	1.9 (1.2-3.0)	0.006
<70 years (51)		
≥70 years (71)		
Tumor-trachea distance	2.2 (1.2-4.2)	0.015
≤ 5mm (21)		
> 5 mm (101)		

Supplementary Materials Figure S1. Progression free survival of patients with tumor size < or ≥ 5 cm (a) and patients with primary lung cancer and tumor size < 5 cm (b).

a. **b.**

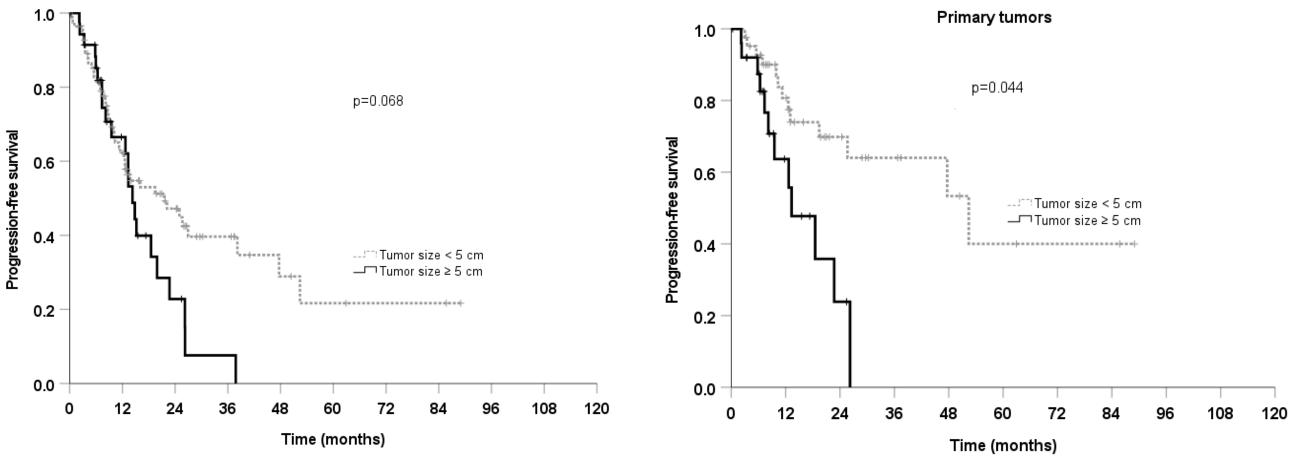

Supplement: Supplementary file 1 [file cancers-14-05908-s001.zip › cancers-2017742-supplementary.pdf]
